# Cooled radiofrequency ablation of the sacroiliac joint a retrospective case series

**DOI:** 10.1186/s12891-023-06344-7

**Published:** 2023-04-04

**Authors:** Joseph Maalouly, Prashanth J Rao

**Affiliations:** Brain and spine surgery, Norwest Private Hospital, Suite G16/9 Norbrik Dr, Bella Vista, NSW 2153 Australia

**Keywords:** Cooled radiofrequency ablation, Sacroiliac joint, Low back pain

## Abstract

**Background:**

Sacroiliac (SI) joint dysfunction is a common source of back pain. Recent evidence from different parts of the world suggest that cooled radiofrequency ablation of sacral nerves supplying the SI joints has superior pain alleviating properties than currently available treatment options for SI joint dysfunction.

**Patients and methods:**

After obtaining institutional review board approval, the medical records of 81 patients who underwent cooled radiofrequency ablation in a single institution and by a single surgeon were analyzed retrospectively. The recurrence of pain, progression to fusion and functional outcomes were noted. The patients were operated on between June 2020 and December 2021, they include 59 females and 22 males, the average age was 55.4 ± 17.3. Follow up was at least 6 months postoperative.

**Results:**

22 of the patients had previously underwent lumbar fusions. Follow up period ranged from 6 to 18 months. After radiofrequency ablation, 7 patients progressed to fusions, and 6 patients had to have the procedure done again to relieve their pain. Student t-test was used to compare between preoperative and postoperative values of NPRS (numerical pain rating score) and ODI (Oswestry disability index). It showed significance with P-value < 0.001 in both.

**Conclusions:**

Sacroiliac joint radiofrequency ablation is a good option in the treatment of SI joint pain showing good results in the short term follow up period. It is a simple procedure that can be done in less than 30 min and is capable of providing significant pain relief for patients with sacroiliac joint dysfunction.

## Introduction

The sacroiliac joint (SIJ) connects the sacrum to the pelvis. Inflammation around that joint from various possible contributing factors such as trauma, pregnancy, and previous lumbar fusion, can cause significant pain and disability [1–3]. Currently, the management of SIJ pain consists of conservative therapy such as physiotherapy and medications [4]. The treatment can be escalated to corticosteroid injections, radiofrequency ablation or fusions [5, 6]. Unfortunately, there is lack of evidence to support one treatment as superior to all others [7]. Conventional radiofrequency ablation had variable degrees of success and it is believed to be due to the smaller treatment area produced with their probes as is demonstrated by multiple studies [8–10]. Thus, this limits their effectiveness to some degree in targeting the SIJ nerves with variable anatomy [11, 12].

Cooled radiofrequency ablation is a relatively new procedure that allows cooling of the probes which can achieve a larger tissue lesion area in comparison with its predecessor. This may in turn lead to better outcomes [13, 14].

Our study has one of the largest patient cohort in the literature, with up to 18 months follow-up using cooled radiofrequency ablation. Our aim is to report our results in terms of functional outcomes, therapy failure and recurrence of pain.

## Patients and methods

After obtaining institutional review board approval, the medical records of 81 Australian patients who underwent cooled radiofrequency ablation in a single institution (Norwest private hospital, NSW) and by a single surgeon were analyzed retrospectively; recurrence of pain, progression to fusion and functional outcomes were noted. The patients were operated on between June 2020 and December 2021. Follow up was at least 6 months, and at most 18 months follow up. Selection criteria include intractable pain of more than 3 months duration, failure of conservative therapy, exclusion of other causes for the pain, positive SIJ provocative maneuvers (such as thigh thrust, Geanslen’s test, FABER test, distraction test, compression test, and sacral thrust), and SIJ intra-articular injection achieving more than 50% relief [1, 2]. Exclusion criteria were younger than 18 years of age, pregnancy, infection around surgical site and immunosuppression.

**Fig. 1:**
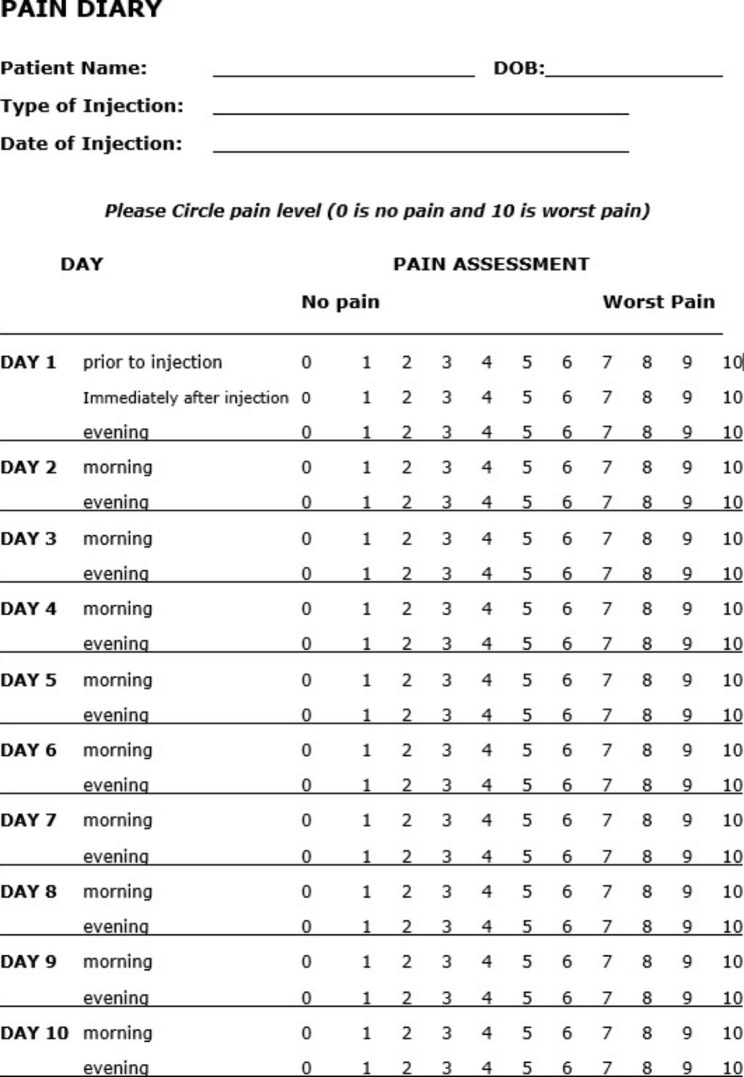
Pain diary

The intra-articular blocks were performed by the surgeon and included local anesthetic 1 mL bupivacaine without epinephrine, and corticosteroid (Dexamethasone 2 mg) injected in both upper and lower pole of SIJ. After the procedure, the patients were given a pain diary (Fig. 1) and reviewed 2 weeks afterwards.

The cooled radiofrequency ablation system used, SInergyTM (Avanos Medical Australia Pty Ltd, Chatswood, Australia), consisted of nine probes that emit a current reaching and maintaining a temperature of 60 degrees Celsius for 2.5 min. The procedure was performed under local anesthesia and sedation using propofol. A high flow nasal cannula therapy was used to assure a good oxygen supply in the prone position. High-flow nasal cannula (HFNC) therapy is an oxygen supply system capable of delivering up to 100% humidified and heated oxygen at a flow rate of up to 60 L per minute. This accomplishes a reduction of nasopharyngeal airway resistance, leading to improved ventilation and oxygenation through the application of a positive pressure environment. In addition to providing positive pressure support to the nasopharynx, a high-flow nasal cannula creates a positive end-expiratory pressure to the lower airways. This effect acts similarly to continuous positive airway pressure support in that it applies a splinting force to keep alveolar airways from collapsing under increased surface tensile stresses during exhalation. Additionally, this allows for improved alveolar recruitment, increasing the effective available surface area within the lungs for gaseous diffusion both to and from the blood. Flow rate normally set at 20 L/min to start and titrated as required. It is now routinely used in theatre for pre-oxygenation of potentially difficult airways and for patients positioned prone for procedures requiring sedation.

During sedation, Propofol TCI (Schneider model) is titrated in 3 mcg/ml with supplemental opioids such as tramadol 50-100 mg and oxycodone2-4 mg plus a small dose of ketamine (20-30 mg). Doses would obviously be titrated to patient comorbidities. The post op medications consisted of Tapentadol IR 50-100 mg Q4hrly PRN.

**Fig. 2:**
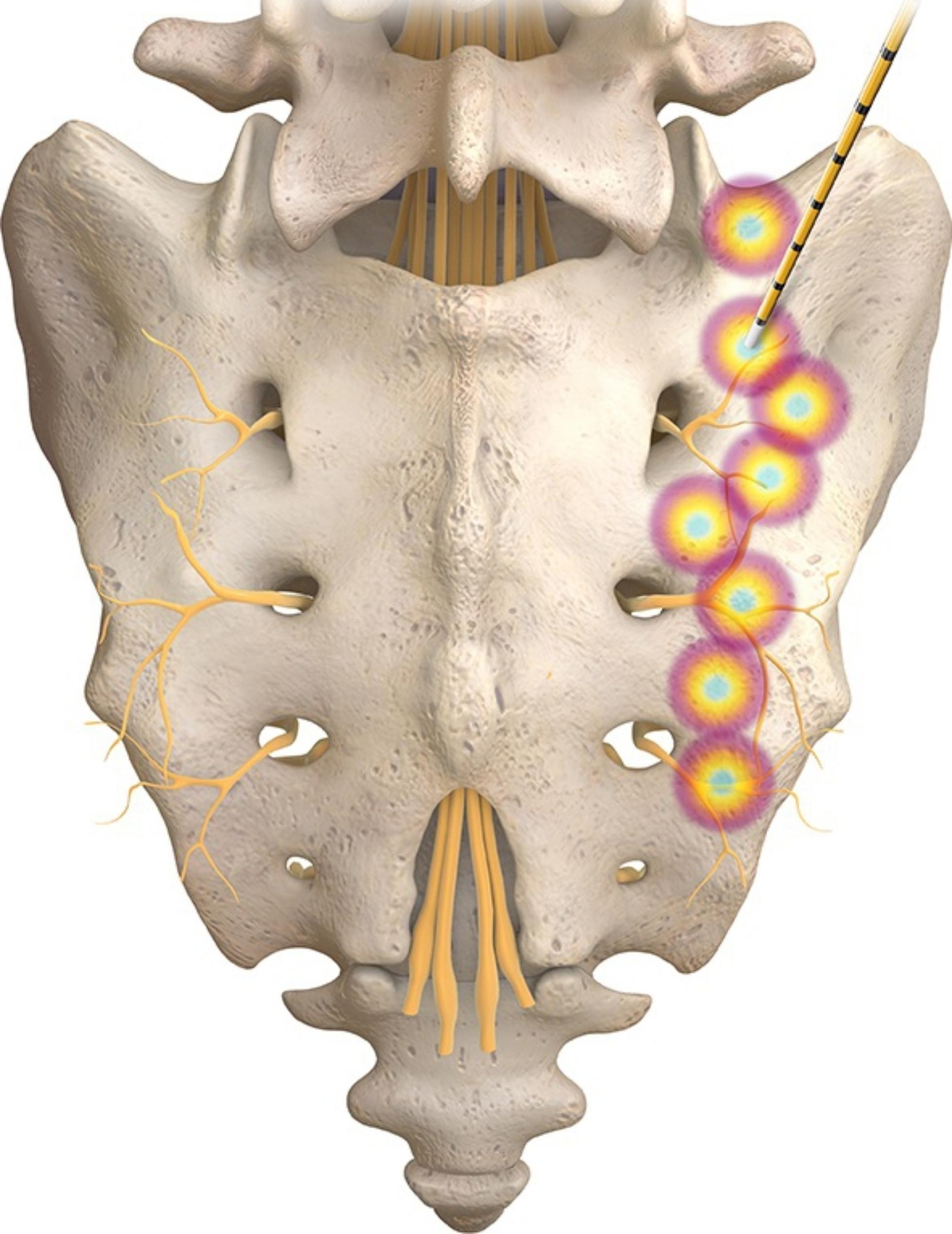
right sacroiliac joint target lesion sites. On the right side, L5 dorsal ramus, 2,4:30 and 6 o’clock for S1 and S2, while S3 only 2, 4:30 o’clock were targeted. (with permission from Avanos)

Eight patients underwent bilateral procedures, while 73 had unilateral ablation. On the right side, 2,4:30 and 6 o’clock for S1 and S2, while S3 only 2, 4:30 o’clock were targeted (Fig. 2). Equivalent targeting on the left side lateral aspect of the foramen was performed. Furthermore, the L5 dorsal ramus on the affected side was targeted as well. Stimulation mode was used to ensure no motor nerves were affected prior to commencing the ablation. one millilitres of mixed local anesthetic, 0.5 mL bupivacaine without epinephrine, and 0.5mL corticosteroid (Dexamethasone 2 mg) were injected at each lesion site.

Data collected included demographics and past history of previous lumbar fusion. Results were considered in terms of relief of pain, recurrence of SIJ pain, and treatment progression to fusion. Pain was evaluated by using the pain diary, NPRS and ODI postoperatively. The provocative maneuvers were used to assess the pain relief.

Statistical analysis consisted of using student t-test in SPSS to compare the preoperative and the postoperative values of functional outcomes scores. (IBM Corp. Released 2016. IBM SPSS Statistics for Windows, Version 24.0. Armonk, NY: IBM Corp)

## Results

Our series of 81 patients included 59 females and 22 males, with an average age of 55.4 ± 17.3 (Table 1). Twenty-two of the patients had previous lumbar fusions.

**Table 1:** patient demographics

Variable	Value
Age	55.44 ± 17.3
Gender	59 F, 23 M
BMI	28.3 ± 4.6

After the procedure, 7 patients progressed to fusion and 6 patients had to have the procedure done again after at least a 9 month’s period to relieve their pain. Progression to fusion was due to failure of conservative management and the presence of a mechanical cause of the dysfunction such as instability or laxity, or overload of the joint by a previous fusion.

Pain relief was observed with the mean NPRS (numerical pain rating score) decreasing from 7.86 ± 1.48 to1.47 ± 1.37 at 1.5 months. Further pain relief was noted after the ODI (Oswestry disability index) score collected at 3 months postoperatively reduced from 43.3 to 10.67. It showed significance with P-value < 0.001 in both (Tables 2 and 3). On average, it took between two and six weeks for the full effect of the ablation to be apparent. Patients’ reported side effects (10%) include buttock soreness, patchy numbness across the buttocks, and neuropathic pain, all of which resolved within 6 weeks.

**Table 2:** NPRS preoperative (during initial consult in the clinic) and 1.5 months postoperative

	NPRS preoperative	NPRS postoperative	P-value
Mean	7.86	1.47	< 0.001
Std. dev.	1.48	1.37	

**Table 3:** ODI preoperative (during initial consult in the clinic) and 3 months postoperative

	ODI preoperative	ODI postoperative	P-value
Mean	43.32	10.67	< 0.001
Std. dev.	13.49	3.06	

## Discussion

This study has one of the largest cohorts in the literature. In our series, most patients were females (72.8%), and there was a high prevalence of previous lumbar fusion (27%). This prevalence is not clear, as a lumbar fusion may increase the presence of SIJ dysfunction, but also many patients may be misdiagnosed, especially those who have facet joint arthrosis and were not clinically examined specifically for SIJ dysfunction. The diagnosis in our patients was made after exclusion of other causes, positive provocative maneuvers and SIJ injection test. The pain significantly improved after the procedure as seen on the functional outcomes scores. Any side effects from the procedure resolved in 6 weeks.

Multiple authors have found significant benefits when using cooled radiofrequency ablation for the treatment of SIJ compared to the traditional method [8, 13, 15]. In a recent meta-analysis, most authors utilized an anesthetic block for the diagnosis of SIJ pain with variability in the pain relief cutoff threshold between 50 and 75% [15]. However, Cohen et al. showed there was no difference in the therapeutic benefit between the thresholds [16].

The posterior sacral network is made up from contributing branches from L4-L5 dorsal rami and S1 to S4 lateral branches [16–19]. Sometimes, even S5 can contribute to this network [18]. The SIJ innervation is subject to a lot of variation and this may lead to disparity in the expected outcomes.

One way to possibly improve the outcomes is to achieve a larger lesion size. In a recently published overview study of cooled radiofrequency ablation, it was noted that a larger lesion likely contributes to more denervation and reduced chance of missing the nerves[20]. The diameter of the area can be 2 mm in unipolar, 6 mm in bipolar radiofrequency and between 8 and 10 mm in cooled radiofrequency ablation [21, 22]. It was recently demonstrated on MRI that lesion size might be affected negatively by the proximity of the probes to bones, which can be an important factor in achieving better treatment results [23].

Furthermore, multiple recently published articles using SInergy™ demonstrate benefit in ODI, NPRS, and visual analog scale scores in these patients [24, 25] In a study by Cohen et al. they used a placebo group and they demonstrated significantly better results with radiofrequency compared to placebo[25]. While Stelzer et al. evaluated cooled radiofrequency ablation in sacroiliac joints in 126 patients with 20 months followup and assessment of functional scores; he noted a significant decrease of opioid use and pain scores [24]. They infiltrated with local anesthetic in both groups. Moreover, a randomized controlled trial compared cooled radiofrequency ablation to placebo and noted significant improvement in quality of life in the ablation group [26]. Additionally, patients with recurring symptoms treated with cooled radiofrequency ablation had significantly more benefits for longer duration the second time [27]. In our study, the use of corticosteroid may have contributed with an additional degree of relief in immediate postoperative period.

As for the secondary effects, the cause of the neuropathic pain and buttock numbness that occurs after ablation is likely due to the middle cuneal nerve, which is formed by sacral lateral branches [28]. The buttock soreness we believe is due to the dissipated heat (60 °C) and inflammatory reaction which causes the muscles to be tense.

The use of HFNC (high flow nasal cannula) has multiple advantages when compared to conventional oxygen therapy including the high-flow rates match the patient’s inspiratory flow rates, deliver a constant FiO2, increase the partial arterial pressure of oxygen (PaO2)/FIO2 ratio, the inhaled heated and humidified gas can improve mucociliary motion and sputum clearance, reduced upper airway resistance, reduced work of breathing and amelioration of thoraco-abdominal synchrony [29].

We recognize that there are some limitations to this study. It is a retrospective review study, with no control groups. There was no traditional radiofrequency group to compare with, thus we cannot make a superiority claim of one modality over another. No serious complications were observed in the patients.

## Conclusion

To conclude, this is one of the largest reported patient cohort showing significant improvement in functional outcomes with cooled radiofrequency ablation for the sacroiliac joint pain. It also notes that the procedure can be repeated with likely increased duration of pain relief.

## Data Availability

The datasets used and analyzed during the current study are available from the corresponding author on reasonable request.

## Abbreviations

NPRS: Numerical pain rating score
ODI: Oswestry disability index
SIJ: Sacroiliac joint
